# Efficient and Cost-Effective Alternative Treatment for Recurrent Urinary Tract Infections and Interstitial Cystitis in Women: A Two-Case Report

**DOI:** 10.1155/2014/698758

**Published:** 2014-12-21

**Authors:** Anthony Mansour, Essa Hariri, Samar Shelh, Ralph Irani, Mohamad Mroueh

**Affiliations:** ^1^School of Medicine, Lebanese American University, Byblos 1401, Lebanon; ^2^School of Pharmacy, Lebanese American University, Byblos 1401, Lebanon; ^3^Clinic Dr. Ralph Irani for Alternative Medicine, Beirut 1202, Lebanon; ^4^Pharmaceutical Science Department, School of Pharmacy, Lebanese American University, Byblos 1401, Lebanon

## Abstract

Urinary tract infections (UTIs) are among the most common bacterial infections affecting women. UTIs are primarily caused by *Escherichia coli*, which increases the likelihood of a recurrent infection. We encountered two cases of recurrent UTIs (rUTIs) with a positive *E. coli* culture, not improving with antibiotics due to the development of antibiotic resistance. An alternative therapeutic regimen based on parsley and garlic, L-arginine, probiotics, and cranberry tablets has been given. This regimen showed a significant health improvement and symptoms relief without recurrence for more than 12 months. In conclusion, the case supports the concept of using alternative medicine in treating rUTI and as a prophylaxis or in patients who had developed antibiotic resistance.

## 1. Introduction

The urinary tract is a sterile environment in healthy people and remains one of the most susceptible sites for bacterial infections [1]. About 50% of women experience a UTI compared to 12% for men, with a recurrence rate of 25% within 6–12 months [2, 3]. An uncomplicated UTI is one that does not progress to a severe disease in a normal host with no functional or anatomical abnormalities of the urinary tract, and its primary treatment is for symptomatic relief [4]. Furthermore, the predominant etiology of uncomplicated UTIs in young women is the uropathogenic* Escherichia coli* (UPEC), representing more than 80% of the cases and increasing the likelihood of a recurrent UTI [3, 5]. Recurrent UTIs are defined as two uncomplicated infections occurring within six months or 3 infections within one year after the treatment and complete resolution of the previous symptomatic infection [6]. Evidently, antibiotics are the most commonly prescribed treatment for UTIs, and those suffering from recurrent UTI are given low prophylactic antibiotic doses [7]. However, a major concern arises due to the increased rates of resistance to treatment of UTIs, which makes their management more challenging while seeking the use of more expensive and less effective drugs [8, 9].

This paper reports two cases of females who were treated with a regimen of dietary supplements after being diagnosed with recurrent UTIs resistant to most antibiotics and showed full healing.

## 2. Case  1

A 28-year-old female presents with a history of rUTI, which started at the age of 11. She suffered symptoms of severe constipation that she treated with herbal laxatives. She experienced episodes of fatigue, pubic pain, loss of appetite, malaise, dysuria, polyuria, urinary incontinence, and macroscopic hematuria. The frequency and severity of the symptoms increased with age, and its recurrence has shifted from occurring once every year to once every 2-3 months after she started having regular sexual intercourse. In addition, she suffered from dyspareunia and urgent urination that were aggravated after tampons usage. Pelvic ultrasound did not show any renal calculi or bladder enlargement, yet it revealed signs of interstitial cystitis.

Different antibiotics were given for treatment including ciprofloxacin (500 mg b.i.d), ofloxacin (200 mg b.i.d) or norfloxacin (400 mg b.i.d), cefuroxime (500 mg b.i.d), or amoxicillin/clavulanic acid (1 g b.i.d). Resistance was developed to these drugs and others were given aiming to prevent the recurrence such as nitrofurantoin (100 mg bid) and fosfomycin (3 g/day), to which she was sensitive. However, the symptoms of interstitial cystitis persisted despite the antibiotic therapy and the patient developed candidiasis that was resolved with fluconazole (150 mg) taken every 5 days after starting antibiotic treatment. Yet the patient became nonresponsive to fluconazole, which was substituted with itraconazole (100 mg bid every 2 days).

One year ago, she started an alternative medical treatment that included simultaneous intake of cranberry tablets (containing 700 mg cranberry extract conc Tit 1%, intake of proanthocyanidin 7 mg, grapefruit seeds dry extract 4/1 100 mg,* Orthosiphon* titrated dry extract 0.2% 100 mg, intake of sinensetin 0.2 mg, and goldenrod titrated dry extract 10/1 100 mg), probiotics (containing vitamin B1 0.42 mg, vitamin B2 0.48 mg, vitamin B6 0.6 mg, niacin 5.4 mg, vitamin B5 1.8 mg, vitamin B12 0.3 *μ*g, and total milk enzymes:* L. rhamnosus*,* L. acidophilus*,* Streptococcus thermophilus*,* Bifidobacterium bifidum*, and* L. bulgaricus*, living cells not less than 2 billion), L-arginine (2 tablets/day, arginine hydrochloride 1000 mg in one serving capsule), and 1 tablet of magnesium at night (magnesium oxide 300 mg in each serving capsule). Following this regimen, the frequency and urgency to urinate decreased and UTI recurred after 2 month. Afterwards, she started taking garlic softgel tablets (serving size 2 softgels: garlic oil 4.6 mg, 500 : 1 concentrate equal to 2300 mg fresh garlic) and parsley seed oil tablets (110 *μ*g, 2000 : 1 concentrate equal to 220 mg fresh parsley). These have shown a decrease in the UTI recurrence to 6-7 months and eventually no recurrence was recorded for the last 12 months. Moreover, the severity of recurrence has decreased and response to antibiotics has become more pronounced. Later when she discontinued garlic intake and became noncompliant to this treatment, UTI reappeared after 2 months.

## 3. Case  2

A 28-year-old female presented with the history of rUTI since the age of 23. The patient's symptoms include burning sensations in the lower area with stabbing-like pain, urgency, and dysuria followed by hesitancy. The patient also reported a high frequency of urination, reaching 10 times/hr. In her first recurrent episode, urinalysis revealed pyuria (8–10 WBCs/hpf) and positive nitrites test and* E. coli* culture. Then, ciprofloxacin was prescribed (500 mg bid for 5 days) and she experienced 2-3 recurrence episodes with positive* E. coli* cultures. Later on, resistance to ciprofloxacin was developed with pelvic echogram and cystoscopy not showing any abnormality, while urinalysis showed persistent bacteriuria and cultures of* E. coli* resistant to fluoroquinolones.

Along with recurrent UTI over the years, interstitial cystitis also started in addition to chronic daily symptoms of overreactive bladder, urgency, frequency, and pubic pain. Nitrofurantoin (2 tablets/day) was prescribed along with fluvoxamine (1 tablet t.i.d.) to alleviate symptoms of overreactive bladder. Her previous history included no sexual activity for the last 4 years, persistent constipation, healthy diet and lifestyle, inadequate water intake, and the use of intimate perfumed washing gel fragranced, panty-liners and synthetic polyester lingerie, and back to front wiping hygiene.

With time the patient entered a depression due to the rUTI with persistent* E. coli* cultures and the chronic interstitial cystitis that remained untreatable. An alternative treatment regimen was prescribed and it included cranberry tablets (1 tablet t.i.d.), probiotics (1 tablet before breakfast and dinner), garlic and parsley (6 tablets at night), magnesium tablets (2 tablets 300 mg each at night), evening primrose oil (2 tablets after lunch), and L-arginine (500 mg, 1 tablet t.i.d.). Several weeks following this regimen, the patient's symptoms including an overreactive bladder diminished, and urgency, frequency, and pubic pain decreased by 80%. Cultures remained negative and the patient experienced only one interstitial cystitis episode, which was alleviated with L-arginine (1000 mg), magnesium tablets, and sustained hydration.

## 4. Discussion

The failure of conventional antibiotic therapy to prevent and treat rUTI due to antibiotic resistance represents a major concern in clinical practice nowadays. Hence, alternative therapy has emerged to help many young women relief their symptoms, prevent the recurrence, and decrease the frequency of bacterial resistance [10]. In this study, we report an effective comprehensive, alternative therapeutic regimen for treating and preventing rUTIs, which are nonresponsive to most commonly used clinical protocols and guidelines.

For women suffering from rUTI, conventional management is based on low-dose antibiotic prophylaxis using trimethoprim-sulfamethoxazole (40 mg/200 mg daily), nitrofurantoin (100 mg/day), or cephalexin (250 mg daily) [11]. However, the use of trimethoprim-sulfamethoxazole is limited to patients with resistance rates not exceeding 20% and without allergy to sulfa drugs [5, 9]. However, these antibiotics have potentially fatal side effects, where nitrofurantoin may lead to respiratory distress and liver injury [9]. Additionally, the FDA has recently issued a new warning on fluoroquinolone intake due to its association with a permanent neuropathy and increased risk of retinal detachment [12]. The problem of antibiotic resistance is also on the rise since women with frequent recurrent infections and symptoms can self-initiate a 3-day regimen of antibiotics therapy without consulting a physician. Due to the increasing resistance of uropathogenic bacteria to different antibiotics, carbapenems and ertapenem remain the last line of defense that requires cautious use and are reserved only to cases of recurrent UTI due to ESBL strains of uropathogenic* E. coli* [13].

Several factors have contributed to the relative success of this treatment regimen. The significance of this regimen is attributed to the fact that the symptoms of UTI disappeared for a longer duration of time compared to previous recurrent UTIs in the described cases. As a result, this regimen may aid in decreasing the use of antibiotics among UTI patients along with the risk for developing resistance to antibiotics. The persistence of bacteriuria in the first case and its absence in the second case could be attributed to the serious compliance to the given regimen of the second patient over the other.

Furthermore, apigenin, the main component of parsley, was shown to possess diuretic effects in addition to anti-inflammatory properties, which are essential in the treatment of UTIs [14, 15]. Garlic in turn is well known for its antibacterial, antifungal, and antiviral properties, which are attributed to diallyl thiosulphate (allicin) and other sulfur containing compounds [16, 17]. Garlic also exhibits antioxidant, anti-inflammatory, and immune-modulatory effects that aid in the treatment of interstitial cystitis [18]. Very few studies have been conducted on the effect of garlic in the treatment of UTIs, but one study has revealed that garlic had a significant effect on attenuating the virulence of* Pseudomonas aeruginosa in vivo*, in an experimental UTI model [19]. Therefore, the combination of garlic oil and parsley in pills could have a synergistic effect on bacterial growth and proliferation. Moreover, cranberry contains significant amounts of D-mannose, which is able to adhere to the bladder epithelium, thus interfering with the adherence of* E. coli* and causing it to simply wash away during urination [20]. The importance of cranberry has been shown in a randomized clinical trial that demonstrated a decrease in UTI recurrence over a period of 6 months [21]. Besides, a recent study examined the use of cranberry pills as prophylaxis of rUTI in women and found that it reduced the recurrence rate to 1.1 per year, and it was the most cost-effective method of all other therapies tested [22].

During sexual activity, the urinary tract may be colonized by bacteria that move from the bowel or vagina [23]. Hence, oral probiotics preparations are used to help repopulate the normal flora in the GI tract. Probiotics are thought to aid the GI tract or vagina to resist invasion and adhesion of uropathogens, regulate intestinal flora, decrease constipation at the right doses, enhance the immune system, and increase the population of* Lactobacillus* that modulate local pH [24]. A Dutch double-blind trial has shown that a preparation containing* Lactobacillus rhamnosus* decreased antibiotic resistance as compared to trimethoprim-sulfamethoxazole [25]. Other studies have shown that* Lactobacillus* preparations restored and maintained the normal flora in women [26], increased the number of lactobacilli [27], improved vaginal health [28], and caused a relief from symptoms of UTI in women [29]. Hence, restoring a balanced microbial flora is essential in the presence of various UTI-promoting factors such as synthetic lingerie, intimate perfumed washing gel, and tampons. Besides, the aforementioned protocol has a component with anti-inflammatory activity, which makes it useful for both UTIs and cystitis.

The oxidation of L-arginine by nitric oxide synthase results in the formation of nitric oxide (NO), which exhibits antibacterial, smooth muscle relaxation, hormone releasing, and immune modulating properties [30]. Furthermore, a high concentration of NO can inhibit the growth of different microorganisms, decrease oxidative stress, and regulate and regenerate bladder uroepithelial cells, as well as alleviating and treating interstitial cystitis [31]. Thus, L-arginine can play an important role as a prophylaxis after treatment of UTIs to prevent the development of new infections as well as the treatment of an overreactive bladder. Alternatively, magnesium produces a double effect since it can aid in reducing constipation, hence decreasing the recurrence frequency, as well as relaxing the smooth muscle of the bladder leading to less frequent urination [10].

In light of the current common clinical outcomes of UTIs, the need for alternative therapeutic modalities to antibiotics has become more crucial, especially for rUTIs. In addition, the increased rate of hospitalization and its costs increases the risk of acquiring ESBL-producing* E. coli* that are resistant to several antibiotics [10]. The current alternative approach is a comprehensive regimen that can work effectively not only in prophylaxis but also in rUTI, due to its anti-inflammatory and bacteriostatic components. Hence, women having a first episode of acute UTI can follow this regimen to prevent future recurrence. Overall, this comprehensive treatment regimen can be initiated whenever symptoms reappear, with positive bacterial cultures, and L-arginine will be used particularly to relieve patients from overreactive bladder without conferring risk of pyelonephritis. This regimen can also be used alone or complementary to conventional treatment modality, and if the symptoms did not resolve within 3 days, patients can continue on a 7-day regimen along with antibiotics to decrease their urinary tract bacterial load.
